# Artificial Intelligence and Tacit Knowledge Integration in Midwifery: Policy Implications for Improving Healthcare Outcomes

**DOI:** 10.1111/inr.70092

**Published:** 2025-08-08

**Authors:** Shoko Takeuchi, Kazumi Kubota, Sachiyo Nakamura

**Affiliations:** ^1^ Department of Nursing Graduate School of Medicine Yokohama City University Yokohama Kanagawa Japan; ^2^ Department of Data Science Shimonoseki City University Shimonoseki City Yamaguchi Japan; ^3^ Department of Healthcare Information Management The University of Tokyo Hospital Bunkyo‐ku Tokyo Japan; ^4^ Health and Global Policy Institute Chiyota‐ku Tokyo Japan

**Keywords:** AI literacy, artificial intelligence, clinical decision support, healthcare ethics, midwifery, perineal trauma prevention, policy integration, tacit knowledge

## Abstract

**Aim:**

To explore the role of artificial intelligence (AI) in capturing tacit midwifery knowledge and its potential to enhance nursing and midwifery practices and policies, and examine how AI tools, such as machine learning (ML) and natural language processing (NLP), can facilitate the integration of midwives’ experiential knowledge into evidence‐based practice and policy.

**Background:**

Midwifery is largely driven by tacit knowledge gained through personal experience. This knowledge, which is crucial to maternal and newborn care, is often underrecognized in clinical policies. AI presents an opportunity to formalize this knowledge, improve healthcare outcomes, and increase the visibility of midwifery within policy frameworks.

**Sources of Evidence:**

A review of recent peer‐reviewed literature focusing on studies related to AI in healthcare, midwifery practices, and AI ethics was conducted. Thirty‐one sources spanning 2015–2025 were reviewed, emphasizing AI applications in clinical settings and midwifery education.

**Discussion:**

AI has the potential to unlock tacit midwifery knowledge, particularly in perineal trauma prevention and clinical decision‐making. AI models can provide real‐time risk assessments and reduce clinical variability by analyzing qualitative data from experienced practitioners. However, ethical concerns, such as data privacy and algorithmic bias, must be addressed in AI integration.

**Conclusion:**

AI offers substantial potential for improving midwifery practices by formalizing tacit knowledge and enhancing decision‐making. However, successful integration requires careful attention to the ethical, governance, and regulatory issues.

**Implications for Nursing Practice and Policy:**

Policy frameworks should encourage the development of AI tools tailored to midwifery, ensuring that midwives are equipped with the necessary training to work with these systems.

## 1 Background

Midwifery, an essential aspect of maternal healthcare, has long been driven by tacit knowledge and insights based on personal experience, intuition, and expertise developed over time. Tacit knowledge, in contrast to formalized evidence‐based medical protocols, remains a cornerstone of clinical decision‐making in midwifery. Tacit knowledge refers to personal experience, intuition, and bodily sensation‐based knowledge, internalized in the mind and often used unconsciously, making its articulation and sharing with others difficult (Sturmberg and Martin 2008). Table 1 shows the characteristics of tacit and explicit knowledge in midwifery practice. The former is based on experience and intuition and is difficult to verbalize, whereas the latter can be documented and shared but cannot fully compensate for delicate judgments and flexible responses in the field. For example, midwives rely on their ability to identify subtle signs of potential perineal trauma, e.g., tissue elasticity or fetal position, during labor management (Lindgren et al. 2011). Moreover, Isacson et al. (2022) reported variations in the management of the third stage of labor and the timing of cord clamping among midwives. These insights are critical to the well‐being of mothers and children but are not always easily transferred into standard clinical guidelines (Kim and Kim 2018).

**TABLE 1:** Comparison of tacit and explicit knowledge in midwifery.

Aspect	Tacit knowledge	Explicit knowledge
Definition	Experience and intuition	Codified, documented information
Process of learning	Acquired through practical experience, observation, and iterative experimentation	Acquired through education, training, and guidelines
Verbalization	Difficult to articulate or formalize	Easy to document and share
Examples in midwifery	Judgment of perineal distensibility through tactile sensation, intuitive perception of fetal rotation tendencies, and assessment of maternal condition changes based on breath sounds and skin color	Quantitative records of labor progression (e.g., cervical dilation, fetal heart rate, estimated blood loss), care protocols, and clinical practice guidelines
Limitations	Difficult to formalize and standardize	Limited in addressing contextual subtleties

In the context of nursing, sharing tacit knowledge plays a considerable role in enhancing clinical decision‐making, particularly when mediated by organizational trust (Yoo et al. 2019). This process resembles the knowledge creation cycle described in the socialization, externalization, combination, and internalization (SECI) model proposed by Nonaka et al. In this model, individuals’ tacit knowledge is transformed into explicit knowledge within an organization, which is then reconverted to tacit knowledge, thereby generating new knowledge (Nonaka et al. 2000). In the context of midwifery practice, the sharing and creation of knowledge based on the SECI model can also contribute to the effective use of tacit knowledge derived from experience.

In Japan, the ongoing decline in birth rates has reduced opportunities for midwives to become involved in childbirth, thereby limiting their ability to develop and accumulate experiential knowledge. In 2023, the total fertility rate in Japan will reach a historic low of 1.2, highlighting the severity of the demographic challenge and further exacerbating the difficulties midwives face in gaining hands‐on experience (Ministry of Health, Labour and Welfare 2023).

With the ongoing digitalization of healthcare systems, artificial intelligence (AI) presents a novel opportunity to formalize tacit knowledge. AI tools, such as machine learning (ML) and natural language processing (NLP), can capture, structure, and apply this unspoken expertise in clinical decision‐making, thus making it accessible and actionable in a standardized form (Seghroucheni et al. 2023). AI technologies have the potential to convert the experience‐based knowledge of midwives into explicit, codified data that can improve the overall quality of midwifery practice and the consistency of patient care (Laderas et al. 2024).

## 2 Aim

This paper aimed to explore how AI can facilitate the integration of tacit midwifery knowledge into evidence‐based practices and healthcare policies. Specifically, it focuses on the application of AI tools such as ML and NLP to enhance midwifery practice, particularly in areas such as perineal trauma prevention, clinical decision‐making, and risk assessment. Additionally, the paper addresses the ethical, regulatory, and governance challenges associated with AI integration in midwifery and provides policy recommendations for the responsible use of these technologies.

## 3 Sources of Evidence

This paper is grounded in a comprehensive review of peer‐reviewed literature on AI applications in healthcare, midwifery, and AI ethics. The literature search was performed using PubMed, MEDLINE, and CINAHL databases. Keywords included midwifery, risk prediction, clinical judgment, perineal trauma, artificial intelligence, machine learning, natural language processing, ethics, and bias. The inclusion criteria comprised studies related to AI application in midwifery practice, education, and healthcare. Articles deemed less relevant for the aims of this study were excluded. Given the rapid advancement of AI technologies and the increasing global discourse on AI ethics (particularly since 2018, reflected in international guidelines such as those issued by the WHO), this review focused on recent studies to gain practical insights. Therefore, the search was limited to studies published between 2015 and 2025. A total of 199 articles were identified through the literature search. Of these, 31 articles covering various aspects of AI integration into midwifery practice, including decision‐support systems, risk prediction, and midwifery education, were included in this review.

### 3.1 Potential and Applications of AI in Midwifery

The potential for AI to transform midwifery practices has been a topic of considerable research. Seghroucheni et al. (2023) reported how NLP and ML can be used to convert tacit knowledge into explicit data, enabling midwives to make informed decisions based on the collective wisdom of their profession. NLP can extract relevant information from clinical notes, interviews, and other unstructured data, which can then be processed by AI to identify patterns and trends. These data‐driven insights can help midwives make more accurate decisions and standardize practices across different settings (Scharp et al. 2024).

The integration of AI into midwifery is exemplified by its role in preventing perineal trauma during childbirth. Hu et al. (2025) developed an AI model that predicts the risk of mediolateral episiotomy, a common intervention for preventing perineal lacerations during childbirth. By analyzing maternal history, fetal positioning, and other clinical data, AI can provide real‐time recommendations to midwives, improving their ability to identify at‐risk patients early and making it easier to take preventive actions. Chill et al. (2021) proposed a personalized prediction algorithm for obstetric anal sphincter injury, demonstrating the capacity of AI to improve outcomes by analyzing clinical data during labor. Furthermore, Borycka et al. (2025) suggested a new diagnostic technique using ML‐assisted impedance spectroscopy to detect obstetric anal sphincter injuries accurately. Their study demonstrated the utility of AI in early identification and intervention, allowing midwives to assess patients in real‐time and improve clinical decision‐making during childbirth.

### 3.2 The Role of AI in Midwifery Education

The value of AI in midwifery education has been demonstrated in the literature. Kranz and Abele (2024) highlighted the use of AI‐powered simulations to train midwifery students. These simulations create realistic scenarios in which students can practice decision‐making and receive feedback on their performance. AI‐driven tools allow greater exposure to various clinical situations and help students develop critical thinking and clinical judgment skills that may not be readily available in traditional clinical settings (Kranz and Abele 2024).

### 3.3 Ethical Considerations in AI Applications

The application of AI in midwifery has ethical considerations. The World Health Organization (2021) has emphasized the importance of transparency, data privacy, and accountability in AI development. Ethical concerns regarding the bias in AI algorithms, especially in predictive models, have also been raised by Obermeyer et al. (2019). If AI systems are trained using biased data, they may perpetuate existing health disparities. It is crucial that AI systems used in midwifery are developed using diverse and representative datasets to ensure equitable outcomes for all patients.

## 4 Discussion

### 4.1 Transforming Tacit Knowledge Into AI‐Driven models

One current international trend is the marginalization of tacit knowledge within practical knowledge. Reinders (2010) argued that although tacit knowledge is a cornerstone supporting professional practice and quality of care, its value is often overlooked because of the increasing emphasis on objective performance evaluations and outcome measures. Tacit knowledge in midwifery is often rooted in intuitive assessments that experienced midwives make based on years of practice. However, this valuable information is often difficult to express in written guidelines or clinical protocols. For example, experienced midwives can detect early signs of perineal trauma using subtle physical cues that are not easily quantifiable (Lindgren et al. 2011). Cioffi (2012) emphasized that unless strategies are urgently developed to document and use the unique tacit knowledge of experienced clinicians, a substantial amount of valuable knowledge may be irretrievably lost. AI provides a promising solution by capturing this unspoken expertise and converting it into actionable data to guide clinical decisions. For example, AI models can assist midwives in real‐time during labor, for instance by identifying patients at risk of perineal lacerations or other complications (Laderas et al. 2024). Moreover, the integration of AI into clinical practice can reduce variability in care and improve the consistency and quality of midwifery practices across settings (Seghroucheni et al. 2023). Therefore, AI usage may allow for consistently high‐quality care provision regardless of the experience level of midwives, potentially contributing to improvements in maternal and child health outcomes.

### 4.2 Ethical Considerations in AI Integration

Although AI offers numerous benefits, it must be integrated into midwifery practice with careful consideration of ethical concerns. One key issue is the privacy and security of patient data. The World Health Organization (2021) guidelines highlight the protection of individual privacy as a major concern in the application of AI in healthcare. Given that midwifery data often involve highly personal and sensitive information, ensuring confidentiality is paramount. Therefore, effective use of AI systems requires strict adherence to data privacy regulations and clear communication with patients regarding data use.

Moreover, transparency is critical to ensure that AI‐driven decisions are trusted and understood by healthcare professionals (Kiseleva et al. 2022). Midwives must interpret AI recommendations and understand how they are created. If AI systems operate in “black boxes,” where the decision‐making process is hidden from users, midwives may be less likely to trust or adopt these tools. Therefore, AI systems must be designed in ways that allow transparency and interpretability, ensuring that midwives can make informed decisions based on AI‐generated insights.

Another important concern is algorithmic bias. If AI systems are trained on biased data, they may produce unfair or inaccurate predictions, particularly for marginalized populations (Obermeyer et al. 2019). For example, midwives predict perineal tear occurrence based on various risk factors, including ethnicity, parity, maternal age, and other factors (Abedzadeh‐Kalahroudi et al. 2019). Therefore, AI systems should be developed using diverse and representative datasets that reflect the demographic diversity of the patient population to mitigate this risk. To illustrate this, the following hypothetical scenario can be considered: AI may sometimes assess a high risk of severe perineal tears during labor and recommend a prophylactic episiotomy on the basis of such data. However, midwives may comprehensively evaluate factors such as perineal elasticity, delivery positioning, and perineal care, including warm compresses and massage, and determine that avoiding an episiotomy could result in less invasiveness and a more natural delivery. Hence, midwives must also be trained to recognize potential biases in AI systems and ensure that these biases do not influence clinical decisions.

By addressing these ethical challenges through thoughtful system design, implementation, and education, AI can be integrated into midwifery practices to enhance the quality of care while safeguarding ethical standards. This will not only build trust in AI technologies among midwives but also ensure that consistent high‐quality care is provided regardless of the midwife's level of experience. Dailah et al. (2024) reported that the integration of AI into nursing and midwifery practices can strengthen decision‐making and improve overall quality of care. Therefore, future research should focus on developing comprehensive frameworks for ethical AI governance in midwifery that can serve as guidelines for policymakers, educators, and practitioners.

### 4.3 Role of Midwifery in AI Development

Midwives must be involved in the design and development of these technologies to ensure that AI systems are aligned with midwifery practices and values. This implicit, experience‐based insight plays a crucial role in clinical and organizational knowledge management. Transforming it into shareable formats is essential for improving professional practices (Seghroucheni et al. 2023). Collaboration among midwives, AI developers, and policymakers is essential for creating AI tools that meet the unique needs of the profession. A recent interview‐based study involving midwives and obstetricians in the United Kingdom reported that transparency and accuracy are essential qualities for AI‐driven cardiotocography to be accepted as a decision‐support tool. Notably, midwives emphasized that AI should serve to complement rather than replace their clinical judgment (Dlugatch et al. 2024). Midwives possess an invaluable knowledge of clinical practice that can guide the development of AI systems, ensuring that these tools enhance their expertise rather than replace them.

Furthermore, midwives must be adequately trained in AI technology. Kranz and Abele (2024) reported that the integration of AI into midwifery education remains in its early stages. They also noted that, as AI becomes increasingly integrated into clinical practice, midwives need to develop digital literacy to interpret AI recommendations and make informed decisions. O'Connor (2022) emphasized the importance of educating students about AI and strongly recommended the implementation of systematic AI literacy education in nursing and midwifery programs.

In addition, Tiase and Cato (2021) argue that AI should be conceptualized not merely as artificial but rather as augmented intelligence, emphasizing the need to redefine AI as a technology that complements and enhances human judgment. This perspective is important for fostering an approach among midwives who view AI not simply as an automation tool, but as an intellectual support reinforcing their professional decision‐making. Moreover, Tiase and Cato (2021) also highlight foundational skills necessary for effective AI use, including an understanding of the data, information, knowledge, and wisdom model, various analytical process types in healthcare, and the three AI branches, noting that “this foundational knowledge is valuable in supporting nursing professionals’ engagement in AI‐related activities.”

Therefore, it is crucial to develop educational programs focusing on AI literacy for midwives to ensure that they are equipped to use these technologies effectively.

## 5 Conclusion

### 5.1 Expanding the Role of AI in Midwifery

AI has tremendous potential for enhancing midwifery practices by formalizing tacit knowledge and improving clinical decision‐making. The integration of AI into midwifery offers numerous benefits, including the ability to standardize care, reduce variability, and improve patient outcomes. By converting tacit knowledge into structured, data‐driven insights, AI can empower midwives to make more informed decisions during labor and delivery, improving the quality of care.

However, the integration of AI into midwifery also raises ethical challenges, such as data privacy, algorithmic bias, and transparency. Addressing these issues and ensuring the safe and effective AI use necessitates urgent and coordinated efforts among stakeholders to establish comprehensive regulatory frameworks and strengthen educational programs.

### 5.2 Future Directions

The future of AI in midwifery is promising; however, to further refine these technologies and ensure their effectiveness in improving clinical outcomes. Promoting AI tool co‐development with the active involvement of midwives and advancing research on unbiased AI models using diverse datasets are essential. In addition, establishing ethical standards to support the appropriate use of AI and enhancing AI literacy education for midwives remain critical challenges moving forward. Further, to ensure sustainable integration and optimization of AI in midwifery practice, longitudinal studies are essential to evaluate the long‐term effects of AI on clinical outcomes, midwifery practice, and patient satisfaction.

### 5.3 Implications for Nursing Practice and Policy

The integration of AI into midwifery and nursing practices has substantial policy implications. To maximize the potential of AI in improving maternal healthcare, comprehensive regulatory frameworks must be established to address data privacy, transparency, and accountability in AI‐driven clinical decision‐making. AI tools should be developed with transparency so that midwives can understand and trust the recommendations.

Midwives must be actively involved in the design and evaluation of AI tools to ensure that these technologies align with their professional knowledge and values. By involving practitioners in the creation of AI systems, we can ensure that AI enhances rather than replaces their expertise in clinical decision‐making. This involvement is essential to ensure that AI systems meet the unique needs of midwifery practices, particularly in areas such as perineal trauma prevention and risk prediction. Moreover, establishing a certification system that verifies the completion of such education is important, ensuring that individuals possess a certain level of knowledge and skills in AI tool operation and associated risk management.

Workforce development is critical. Midwifery and nursing education programs should include digital literacy and AI‐specific training to prepare future practitioners to work effectively with AI tools. Continuous professional development programs focusing on AI literacy should be implemented to ensure that practicing midwives are well equipped to integrate AI into their clinical practice.

Ethical concerns, such as algorithmic bias and data privacy, must be prioritized in policy discussions. Additionally, AI systems must be developed using diverse and representative data to avoid perpetuating existing health disparities. Policies must also ensure that AI tools are used in ways that uphold midwives’ core values of personalized, compassionate care; for example, by establishing review boards that include practicing midwives to evaluate AI integration in clinical workflows. Moreover, professional organizations (e.g., nursing and midwifery associations) should take a leading role in jointly developing guidelines on ethical and safe AI application and conducting AI operation audits, thereby ensuring transparency and social trust. Several initiatives have already been reported as concrete examples. The NHS AI Team from the UK supports projects that facilitate collaboration between technology developers and clinical staff, promoting the development of user‐centered and clinically relevant AI solutions (NHS England 2025). Furthermore, The Nursing and Midwifery Council (2025), the UK regulatory body for nursing and midwifery, plans to revise its Code of Conduct as part of its 2025–2026 corporate plan to ensure that midwives can use AI safely and effectively.

Finally, Goodman et al. (2023) emphasized that AI tools must be continuously evaluated for their impact on patient outcomes and safety to ensure that they contribute to the improvement of clinical practice and align with midwifery goals. This includes monitoring the effectiveness of AI‐driven recommendations and ensuring that they contribute to the improvement of maternal health outcomes. Such policy measures will be the key to enhancing the quality and safety of AI use in future midwifery practices.

## Author Contributions

Conceptualization and methodology: ST, KK, and SN. Data collection: ST and KK. Manuscript writing (Draft): KK. Manuscript writing (critical revisions for important intellectual content): ST and SN. ST and KK are equal contributors to this work.

## Conflict of Interest

The authors declare no conflicts of interest.
